# An enhanced EWMA chart with variable sampling interval scheme for monitoring the exponential process with estimated parameter

**DOI:** 10.1038/s41598-024-58675-7

**Published:** 2024-04-04

**Authors:** Yajie Bai, Jyun-You Chiang, Wen Liu, Zhengcheng Mou

**Affiliations:** School of Statistics, Southwestern University of Finance and Economics, Chengdu, 611130 China

**Keywords:** Exponential process, Estimated parameter, Exponentially weighted moving average, Variable sampling interval, Markov chain method, Optimization algorithm design, Engineering, Mathematics and computing

## Abstract

Control charts have been used to monitor product manufacturing processes for decades. The exponential distribution is commonly used to fit data in research related to healthcare and product lifetime. This study proposes an exponentially weighted moving average control chart with a variable sampling interval scheme to monitor the exponential process, denoted as a VSIEWMA-exp chart. The performance measures are investigated using the Markov chain method. In addition, an algorithm to obtain the optimal parameters of the model is proposed. We compared the proposed control chart with other competitors, and the results showed that our proposed method outperformed other competitors. Finally, an illustrative example with the data concerning urinary tract infections is presented.

## Introduction

The control chart is one of the important tools in statistical process control, mainly used to detect process shifts in the manufacturing process. Shewhart^1^ first proposed a control chart, which effectively detects large shifts. Subsequently, the memory control charts, such as the CUSUM^2,3^ and EWMA^4,5^ control charts, were developed to detect moderate and small shifts. Most recent studies have designed the charts based on the two assumptions. The first assumption is that the quality characteristic follows a normal distribution. The second assumption is that the in-control process parameter is known. When the normal distribution assumption is violated, it may lead to a high false alarm rate for the in-control process^6^. Therefore, many non-normal control charts have been proposed^7–9^. Additionally, in real applications, process parameters are often unknown and need to be estimated. Considering the impact of parameter estimation on control charts, some studies have focused on designing control charts with estimated parameters^10,11^.

In practical applications, the exponential distribution is usually used to fit non-normal distribution data, such as lifetimes or failure times of product, disease infection rate, etc.^12,13^. Consequently, many studies have been conducted on control charts for the exponential processes. For example, Xie et al.^12^ and Zhang et al.^14^ developed the Shewhart-type control charts for the exponential process. Besides, the CUSUM and EWMA-typed charts have been employed to monitor the exponential process^15,16^. However, most studies on monitoring the exponential processes focused on assuming the process parameter is known. In practice, the process parameter needs to be estimated, so the control chart with an estimated parameter should be designed. In addition, due to the asymmetry of the exponential distribution, the performance measure of the two-sided control chart is biased. The unbiasedness refers to the values of the in-control performance measure being consistently greater than that for the out-of-control state^17^. To avoid this bias, one-sided charts are used to monitor the exponential process^18^. However, as we know, a two-sided chart can display both upward and downward shifts on the same chart. Therefore, the two-sided chart is also necessary.

The control charts mentioned in the above literature are all standard fixed-parameter control charts. When sample size, sampling intervals, or other control chart components vary, the chart is referred to as the adaptive control chart. The adaptive control charts are superior to the standard fixed-parameter control charts in monitoring small and medium shifts^19^. Sabahno et al.^20^ categorized adaptive charts into four types: VSS (Variable Sample Sizes)^21^, VSI (Variable Sampling Intervals)^22,23^, VSSI (Variable Sample Sizes and Sampling Intervals)^24^, and VP (Variable Parameters, if all of the chart parameters are allowed to vary)^25^. In this paper, we consider using VSI scheme to construct the proposed control chart for the following reasons: (1) Aykroyd et al.^26^ highlighted the VSI scheme as a recent research hotspot through bibliometric analysis. (2) While much VSI research are based on the normal distributions^27,28^, some studies have extended its applicability to the non-normal distributions^24,29^. However, no studies have yet applied the VSI scheme to monitoring the exponential processes. (3) Similar to studies by Liu et al.^16^, Santiago et al.^31^, Aslam et al.^32^, etc., this research employs transformed the exponential data with a sample size of 1 as the quality characteristic to be monitored, rendering the VSI scheme suitable for use.

To sum up, the exponential distribution is essential in fitting skewed data. The process parameter is unknown and needs to be estimated in practice. Additionally, the performance measure is usually biased when the two-sided chart monitors skewed distributed data. Therefore, it is necessary to design an efficient control chart with unbiased properties and parameter estimation to monitor the exponential process. The main contributions of this study are as follows:

1. Designed a two-sided VSI EWMA control chart to monitor the exponential process with unknown parameter.
2. Derived the transition probability matrix of the proposed VSIEWMA-exp control chart, enabling the Markov chain method to be used to calculate the performance measures of the control chart.
3. Taking inspiration from Yeong et al.^28^, we propose an optimization algorithm for unknown parameters. This algorithm aims to achieve optimal out-of-control detection efficiency at different shift levels while ensuring average in-control performance. Moreover, the performance measure of the optimized VSIEWMA-exp chart is unbiased.

The remainder of this paper is organized as follows: Section "Structural design of the proposed control chart" introduces the proposed VSIEWMA-exp chart control chart. In Section "Investigation of performance measures for the proposed scheme", performance measures are investigated using the Markov chain method. Section "Optimization algorithm design for model enhancement" introduces the optimization algorithm for adjusting model parameters. Additionally, a numerical comparison is presented in Section "Comparison of proposed and existing schemes". A real data on urinary tract infections is used to demonstrate the proposed control chart in Section "Implementation of the proposed schemes". Finally, the concluding remarks are given in Section "Summary remarks".

## Structural design of the proposed control chart

Let $X=\{{\text{X}}_{1},{\text{X}}_{2},\dots \}$ be a random variable following an exponential distribution with the scale parameter $\eta$, denoted as ${X}_{i}\sim \mathit{exp}\left(\eta \right)$, where $i=\mathrm{1,2},\cdots$. The probability density function (pdf) of the exponential distribution is

1 $$f\left(x\right)=\frac{1}{\eta }{e}^{-\frac{x}{\eta }}, x>0, \eta >0.$$

**Figure 1:**
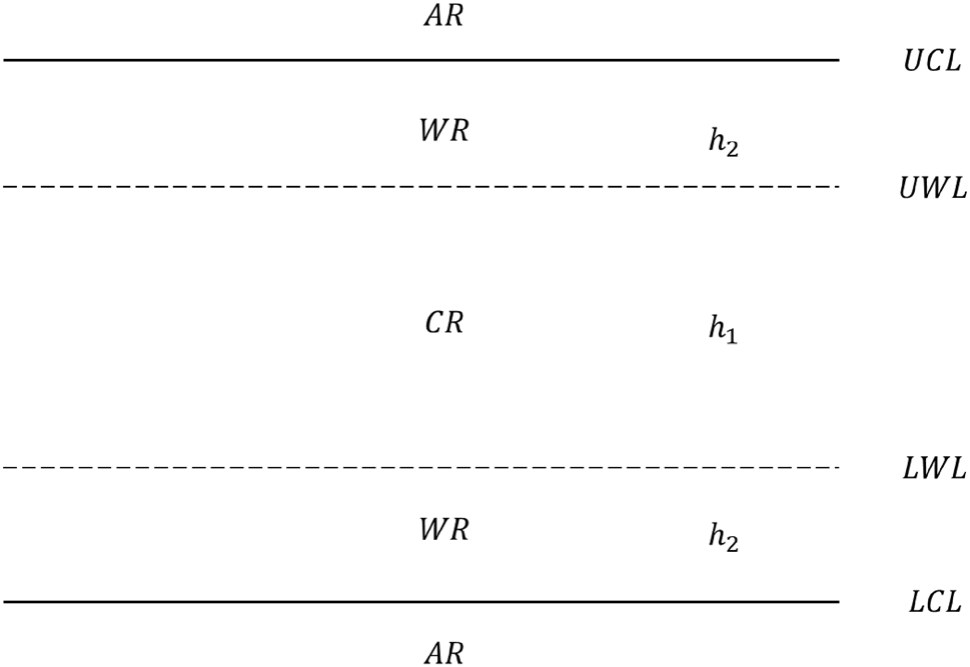
The VSIEWMA-exp chart with action and warning control limits.

This study uses the two-sided chart to design the proposed control chart. The null and alternative hypotheses are presented as follows:

$${H}_{0}: {\eta }_{1}={\eta }_{0}; {H}_{1}: {\eta }_{1}\ne {\eta }_{0},$$

where ${\eta }_{0}$ and ${\eta }_{1}$ represent the scale parameter of the exponential distribution for the in-control and out-of-control states. Let $\delta ={\eta }_{1}/{\eta }_{0}$, which represents the magnitude of the shift. $0< \delta <1$ and $\delta >1$ represent downward and upward shifts, respectively. Let $Y={X}^{1/3.6}$; the advantage of such transformation^30^ is to make the data asymptotically symmetric, thereby using symmetric control limits. Many studies have adopted this statistic^16,31,32^. It is evident that $Y$ follows a Weibull distribution with a scale parameter of ${\eta }_{0}^{1/3.6}$ and a shape parameter of 3.6, denoted by $Y\sim Weibull\left({\eta }_{0}^{1/3.6},3.6\right)$. Next, the EWMA statistic is shown as follows:

2 $${Z}_{i}=\lambda {Y}_{i}+\left(1-\lambda \right){Z}_{i-1}, i=\mathrm{1,2},\dots ,$$

where $\lambda$ is the smoothing parameter with a range of $(0,1]$. This study employs the variable sampling interval (VSI) scheme, where ${h}_{1}$ and ${h}_{2}$ represent the longer and shorter sampling intervals, respectively. When samples are within the central region ($CR$), indicating a low risk of process shift, ${h}_{1}$ is utilized; conversely, when samples fall within the warning region ($WR$), indicating a higher shift risk, ${h}_{2}$ is employed, as shown in Fig. 1. Based on the control chart theory, the definitions of $LCL$, $UCL$, $LWL$, and $UWL$ are as follows:

$$LCL={\mu }_{0}\left(Z\right)-K{\sigma }_{0}\left(Z\right),$$

$$UCL={\mu }_{0}\left(Z\right)+K{\sigma }_{0}\left(Z\right),$$

$$LWL={\mu }_{0}\left(Z\right)-W{\sigma }_{0}\left(Z\right),$$

and

$$UWL={\mu }_{0}\left(Z\right)+W{\sigma }_{0}\left(Z\right),$$

where ${\mu }_{0}\left(Z\right)$ and ${\sigma }_{0}\left(Z\right)$ represent the mean and standard deviation of the statistic $Z$ when the process is in-control, and $K$ and $W$ are model parameters to be optimized in section "Investigation of performance measures for the proposed scheme". Equation (2) can be equivalently written as a moving average of the current and past observations: ${Z}_{i}=\lambda {\sum }_{j=0}^{i-1}{\left(1-\lambda \right)}^{j}{Y}_{i-j}+{\left(1-\lambda \right)}^{i}{Z}_{0}$, where the initial value ${Z}_{0}$ is often taken to be the target value or the process mean. Then, if the ${Y}_{i}$ are independent and have a common standard deviation ${\sigma }_{0}\left(Y\right)$, we have

$${\mu }_{0}\left(Z\right)={\mu }_{0}\left(Y\right),$$

$${\sigma }_{0}\left(Z\right)=\sqrt{\frac{\lambda }{2-\lambda }\left[1-{\left(1-\lambda \right)}^{2i}\right]}{\sigma }_{0}\left(Y\right).$$

Hence, Eqs. (3), (4), (5) and (6) are easily derived accordingly.

3 $${LCL}_{i}={\mu }_{0}\left(Y\right)-K\sqrt{\frac{\lambda }{2-\lambda }\left[1-{\left(1-\lambda \right)}^{2i}\right]}{\sigma }_{0}\left(Y\right),$$

4 $${UCL}_{i}={\mu }_{0}\left(Y\right)+K\sqrt{\frac{\lambda }{2-\lambda }\left[1-{\left(1-\lambda \right)}^{2i}\right]}{\sigma }_{0}\left(Y\right),$$

5 $${LWL}_{i}={\mu }_{0}\left(Y\right)-W\sqrt{\frac{\lambda }{2-\lambda }\left[1-{\left(1-\lambda \right)}^{2i}\right]}{\sigma }_{0}\left(Y\right),$$

and

6 $${UWL}_{i}={\mu }_{0}\left(Y\right)+W\sqrt{\frac{\lambda }{2-\lambda }\left[1-{\left(1-\lambda \right)}^{2i}\right]}{\sigma }_{0}\left(Y\right),$$

where ${\mu }_{0}\left(Y\right)$ and ${\sigma }_{0}\left(Y\right)$ are equal to

7 $${\mu }_{0}\left(Y\right)={\widehat{\eta }}_{0}^{\frac{1}{3.6}}\Gamma \left(1+\frac{1}{3.6}\right),$$

and

8 $${\sigma }_{0}\left(Y\right)={\widehat{\eta }}_{0}^{\frac{1}{3.6}}\sqrt{\left(\Gamma \left(1+\frac{2}{3.6}\right)-{\Gamma }^{2}\left(1+\frac{1}{3.6}\right)\right)}.$$

${\widehat{\eta }}_{0}=\sum_{j=1}^{m}{X}_{j}/m$ is the estimated process parameter, where $m$ denotes the number of samples for the in-control state. Note that as $i$ increases, the term ($1-{\left(1-\lambda \right)}^{2i}$) converges to unity. Thus, these limits converge toward constant levels given as

9 $$LCL={\widehat{\eta }}_{0}^{\frac{1}{3.6}}lcl,$$

10 $$UCL={\widehat{\eta }}_{0}^{\frac{1}{3.6}}ucl,$$

11 $$LWL={\widehat{\eta }}_{0}^{\frac{1}{3.6}}lwl,$$

and

12 $$UWL={\widehat{\eta }}_{0}^{\frac{1}{3.6}}uwl,$$

where

13 $$lcl=\Gamma \left(1+\frac{1}{3.6}\right)-K\sqrt{\frac{\lambda }{2-\lambda }\left(\Gamma \left(1+\frac{2}{3.6}\right)-{\Gamma }^{2}\left(1+\frac{1}{3.6}\right)\right)},$$

14 $$ucl=\Gamma \left(1+\frac{1}{3.6}\right)+K\sqrt{\frac{\lambda }{2-\lambda }\left(\Gamma \left(1+\frac{2}{3.6}\right)-{\Gamma }^{2}\left(1+\frac{1}{3.6}\right)\right)},$$

15 $$lwl=\Gamma \left(1+\frac{1}{3.6}\right)-W\sqrt{\frac{\lambda }{2-\lambda }\left(\Gamma \left(1+\frac{2}{3.6}\right)-{\Gamma }^{2}\left(1+\frac{1}{3.6}\right)\right)},$$

and

16 $$uwl=\Gamma \left(1+\frac{1}{3.6}\right)+W\sqrt{\frac{\lambda }{2-\lambda }\left(\Gamma \left(1+\frac{2}{3.6}\right)-{\Gamma }^{2}\left(1+\frac{1}{3.6}\right)\right)},$$

Next, the VSIEWMA-exp control chart works as follows:

*Step 1:* Collect $m$ in-control samples and estimate scale parameter ${\eta }_{0}$.

*Step 2:* Calculate the control and warning limits based on the optimal model parameters obtained from the proposed optimization model later.

*Step 3:* Let $i=i+1$, draw a random sample ${X}_{i}$ with the sampling interval ${h}_{1}$, and transform it to ${{X}_{i}}^{1/3.6}$. Then, calculate the statistic ${Z}_{i}$.

*Step 4:* If ${Z}_{i}\in CR$, go to Step 3. If ${Z}_{i}\in WR$, go to Step 5. Otherwise, go to Step 6.

*Step 5:* Let $i=i+1$, draw a random sample ${X}_{i}$ with the sampling interval ${h}_{2}$, and calculate the statistic ${Z}_{i}$. Then, go to Step 4.

*Step 6:* If ${Z}_{i}\in AR$, stop the process and eliminate assignable causes.

## Investigation of performance measures for the proposed scheme

In this section, we evaluate the performance of the proposed chart using the conditional average time to signal ($CATS$), which depends on the estimated process parameter ${\widehat{\eta }}_{0}$. $CATS$ is computed utilizing the Markov chain method^33^.

The in-control region $\left[LCL,UCL\right]$ is divided into $2N+1$ discrete subintervals. A larger $N$ indicates a more accurate result. However, as $N$ increases, the computation time also grows, and typically, $2N+1$ is taken to be 50 or greater. Saccucci et al.^34^ consider $2N+1=83$ to be sufficient. The width of the subinterval is $d=(UCL-LCL)/(2N+1)$. ${L}_{j}=LCL+\left[(j-1)(UCL-LCL)\right]/\left(2N+1\right)$ and ${U}_{j}=LCL+\left[j(UCL-LCL)\right]/\left(2N+1\right)$ indicate the lower and upper bounds of the ${j}^{th}$ discrete subinterval, where $j=1,...,2N+1$. Let ${l}_{j}=lcl+\left[(j-1)(ucl-lcl)\right]/\left(2N+1\right)$ and ${u}_{j}=lcl+\left[j(ucl-lcl)\right]/\left(2N+1\right)$, then, we have ${L}_{j}={\widehat{\eta }}_{0}^{1/3.6}{l}_{j}$ and ${U}_{j}={\widehat{\eta }}_{0}^{1/3.6}{u}_{j}$. ${M}_{j}={L}_{j}+\left({U}_{j}-{L}_{j}\right)/2$ represents the midpoint of the ${j}^{th}$ discrete subinterval. It can be rewritten as ${M}_{j}={\widehat{\eta }}_{0}^{1/3.6}\left({l}_{j}+\left({u}_{j}-{l}_{j}\right)/2\right)={\widehat{\eta }}_{0}^{1/3.6}{m}_{j}$. Naturally, the transition probability is equal to

17 $$\begin{gathered} \hat{P}_{kj} \left( {\delta |\hat{\eta }_{0} } \right) = P\left( {L_{j} < Z_{i} < U_{j} {|}Z_{i - 1} = M_{k} } \right) \hfill \\ = P\left( {L_{j} < \lambda Y_{i} + \left( {1 - \lambda } \right)Z_{i - 1} < U_{j} {|}Z_{i - 1} = M_{k} } \right) \hfill \\ = P\left( {\left( {\frac{{\hat{\eta }_{0} /\eta_{0} }}{\delta }} \right)^{{\frac{1}{3.6}}} \frac{{l_{j} - \left( {1 - \lambda } \right)m_{k} }}{\lambda } < \left( {\frac{{X_{i} }}{{\eta_{1} }}} \right)^{{\frac{1}{3.6}}} < \left( {\frac{{\hat{\eta }_{0} /\eta_{0} }}{\delta }} \right)^{{\frac{1}{3.6}}} \frac{{u_{j} - \left( {1 - \lambda } \right)m_{k} }}{\lambda }} \right) \hfill \\ = F_{WB} \left( {\left( {\frac{\gamma }{\delta }} \right)^{{\frac{1}{3.6}}} \frac{{u_{j} - \left( {1 - \lambda } \right)m_{k} }}{\lambda };1,3.6} \right) - F_{WB} \left( {\left( {\frac{\gamma }{\delta }} \right)^{{\frac{1}{3.6}}} \frac{{l_{j} - \left( {1 - \lambda } \right)m_{k} }}{\lambda };1,3.6} \right), \hfill \\ \end{gathered}$$

where ${F}_{WB}\left(\cdot ;\mathrm{1,3.6}\right)$ represents the Weibull distribution with the scale parameter of 1 and the shape parameter of 3.6. Here, $\gamma ={\widehat{\eta }}_{0}/{\eta }_{0}$ follows a Gamma distribution, denoted by $\gamma \sim Gamma(m,1/m)$, where $1/m$ and $m$ represent the corresponding scale and shape parameters, respectively. Then, the transition probability matrix of this Markov chain is given as follows:

$$\widehat{P}\left(\delta |{\widehat{\eta }}_{0}\right)=\left[\begin{array}{cc}\widehat{Q}& \widehat{{\varvec{r}}}\\ {0}{\prime}& 1\end{array}\right]$$

18 $$=\left[\begin{array}{ccccc}{\widehat{p}}_{\mathrm{0,0}}& {\widehat{p}}_{\mathrm{0,1}}& \cdots & {\widehat{p}}_{\mathrm{0,2}N}& 1-{\widehat{p}}_{\mathrm{0,0}}-{\widehat{p}}_{\mathrm{0,1}}-\dots -{\widehat{p}}_{\mathrm{0,2}N}\\ {\widehat{p}}_{\mathrm{1,0}}& {\widehat{p}}_{\mathrm{1,1}}& \cdots & {\widehat{p}}_{\mathrm{1,2}N}& 1-{\widehat{p}}_{\mathrm{1,0}}-{\widehat{p}}_{\mathrm{1,1}}-\dots -{\widehat{p}}_{\mathrm{1,2}N}\\ \vdots & \vdots & \vdots & \vdots & \vdots \\ {\widehat{p}}_{2N,0}& {\widehat{p}}_{2N,1}& \cdots & {\widehat{p}}_{2N,2N}& 1-{\widehat{p}}_{2N,0}-{\widehat{p}}_{2N,1}-\dots -{\widehat{p}}_{2N,2N}\\ 0& 0& \cdots & 0& 1\end{array}\right].$$

The submatrix $\widehat{Q}$ is a $(2N+1) \times (2N+1)$ matrix of transition probabilities for the transient states, while $\widehat{{\varvec{r}}}$ is a $(2N+1) \times 1$ vector satisfying $\widehat{{\varvec{r}}}=(1-\widehat{Q})1$, where $1={(\mathrm{1,1},\dots ,1)}^{\boldsymbol{^{\prime}}}$. Inspired by Saccucci et al.^22^, we compute $CATS$ as follows:

19 $${CATS}_{s}\left(\delta |{\widehat{\eta }}_{0}\right)={b}^{\boldsymbol{^{\prime}}}{\left({\varvec{I}}-\widehat{Q}\left(\delta |{\widehat{\eta }}_{0}\right)\right)}^{-1}{\varvec{h}}, s=\left\{\begin{array}{c} 0,\,\delta =1 \\ 1,\,\delta \ne 1\end{array}\right.,$$

where $b$ is a $(2N+1)\times 1$ vector of initial probability, defined as $$b=\left\{\begin{array}{c}1 {Z}_{i}\in [{L}_{j},{U}_{j}] \\ 0 {Z}_{i}\notin [{L}_{j},{U}_{j}]\end{array}\right.$$.

${\varvec{I}}$ is the identity matrix. ${\varvec{h}}=({h}_{1}^{*},{h}_{2}^{*},\dots {h}_{2N+1}^{*})$ is a $(2N+1)\times 1$ vector, where each element satisfies the following conditions: for ${M}_{k}\in CR$, ${h}_{i}^{*}={h}_{1}$, and for ${M}_{k}\in WR$, ${h}_{i}^{*}={h}_{2}$.

As we know, the $CATS$ varies with ${\widehat{\eta }}_{0}$. Therefore, the unconditional measure $AATS$ is required to assess the chart’s average performance. The $AATS$ is calculated as follows:

20 $${AATS}_{s}\left(\delta \right)=\int {CATS}_{s}\left(\delta |{\widehat{\eta }}_{0}\right)f\left(\gamma \right)d\gamma , s=\left\{\begin{array}{c} 0,\,\delta =1 \\ 1,\,\delta \ne 1\end{array}\right..$$

## Optimization algorithm design for model enhancement

In this section, we propose an optimization algorithm to adjust the model parameters $(\lambda ,K,W,{h}_{1},{h}_{2})$. Our goal is to enhance detection efficiency across various shift levels while ensuring the in-control average performance.

When the process parameter is known, Yeong et al.^28^ proposed an optimization algorithm for optimizing model parameters. Inspired by Yeong et al.^28^, we propose an optimization algorithm for the scenario where the process parameter ${\eta }_{0}$ is unknown. Additionally, the sampling intervals (${h}_{1}$ and ${h}_{2}$) are not predetermined like Yeong et al.^28^, but obtained through model optimization. The optimal model parameters (${\lambda }^{*},{K}^{*},{W}^{*},{{h}_{1}}^{*},{{h}_{2}}^{*}$) are obtained as follows:

21 $$\begin{gathered} \left( {\lambda^{*} ,K^{*} ,W^{*} ,h_{1}^{*} ,h_{2}^{*} } \right) \hfill \\ = \arg \mathop {\min }\limits_{{\left( {\lambda ,K,W,h_{1} ,h_{2} ,m} \right)}} AATS_{1} \left( {\lambda ,K,W,h_{1} ,h_{2} ,\delta ,m} \right) \hfill \\ {\text{Subject}}\,{\text{to}}\,{\text{the}}\,{\text{constraints}} \hfill \\ AATS_{0} = \tau \hfill \\ AASI_{0} = h_{0} , \hfill \\ \end{gathered}$$

The performance measures $A{ATS}_{0}$ and $A{ATS}_{1}$ correspond to in-control and out-of-control states, respectively, calculated using Eq. (20). ${AASI}_{0}$ represents the in-control average sampling interval, computed as ${AASI}_{0}=AASI\left(\delta =1\right)=\int CASI\left(\delta =1|{\widehat{\eta }}_{0}\right)f\left(\gamma \right)d\gamma$, where $CASI\left(\delta =1|{\widehat{\eta }}_{0}\right)={p}_{1}{h}_{1}+{p}_{2}{h}_{2}$ denotes the conditional average sampling interval for the in-control state, with ${p}_{1}$ and ${p}_{2}$ representing the probabilities of using long and short sampling intervals (${h}_{1}$ and ${h}_{2}$). $\tau$ is the specified value of ${AATS}_{0}$, set at 370.4 in this study, and ${h}_{0}$ is the given value of average sampling interval (${h}_{2}<{h}_{0}<{h}_{1}$). Without loss of generality, we set ${h}_{0}=1$.

Here are the steps for the model optimization algorithm we provide, please refer to the supplementary file for the corresponding R code.

*Step 1*: Specify $\delta$ and $m$.

*Step 2*: Set $${h}_{2}=0.1$$, $${h}_{1}={h}_{0}+0.1$$.

*Step 3*: Set $\lambda =0.03$. Solve for $K$ and $W$ based on the constraints ${AATS}_{0}=\tau$ and $A{ASI}_{0}={h}_{0}$.

Step 4: Compute ${AATS}_{1}$ using Eq. (20).

*Step 5*: Increment $\lambda$ by 0.01 while maintaining ${h}_{1}$ and ${h}_{2}$. Repeat Steps 3–4 until $\lambda =1$.

*Step 6*: Increment ${h}_{1}$ by 0.1 while maintaining ${h}_{2}$. Repeat Steps 3–5 until ${h}_{1}=2.5$.

*Step 7*: Increment ${h}_{2}$ by 0.1. Repeat Steps 3–6 until ${h}_{2}=0.9$.

*Step 8*: Terminate the loop and obtain the optimal model parameters $\left({\lambda }^{*},{K}^{*},{W}^{*},{{h}_{1}}^{*},{{h}_{2}}^{*}\right)$ corresponding to the smallest $A{ATS}_{1}$.

## Comparison of proposed and existing schemes

**Figure 2:**
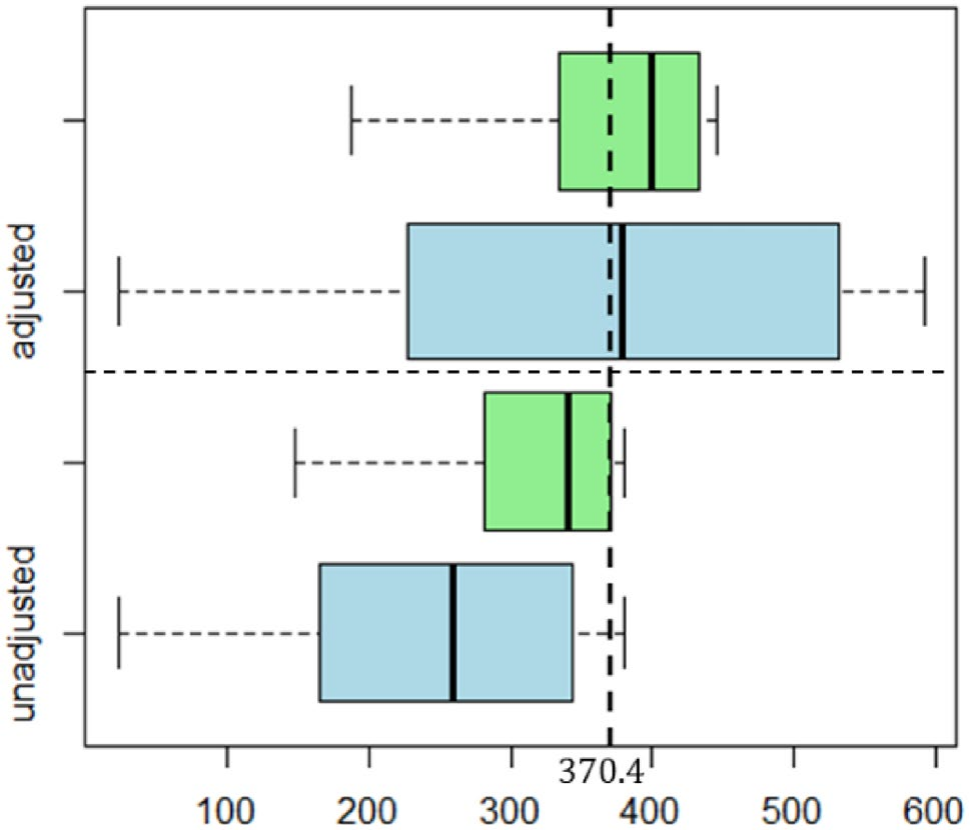
The distribution of ${CATS}_{0}$ for adjusted and unadjusted model parameters. (${ATS}_{0}=370.4$). The green and blue boxplots correspond to scenarios where $m=50$ and $m=200$, respectively.

We present boxplots of ${CATS}_{0}$ for different values of $m$ ($m=\mathrm{50,200}$) in Fig. 2. “unadjusted” refers to ${CATS}_{0}$ is calculated using model parameters based on the assumption of known ${\eta }_{0}$, while “adjusted” indicates ${CATS}_{0}$ calculated using adjusted model parameters optimized through the optimization model detailed in section "Optimization algorithm design for model enhancement". Notably, “unadjusted” yields ${CATS}_{0}$ values mostly below 370.4, indicating a higher false alarm rate. Conversely, the “adjusted” scenario shows improved ${CATS}_{0}$ values. Therefore, the effect of the estimated parameter on ${CATS}_{0}$ was mitigated when using the optimal model parameters.

**Figure 3:**
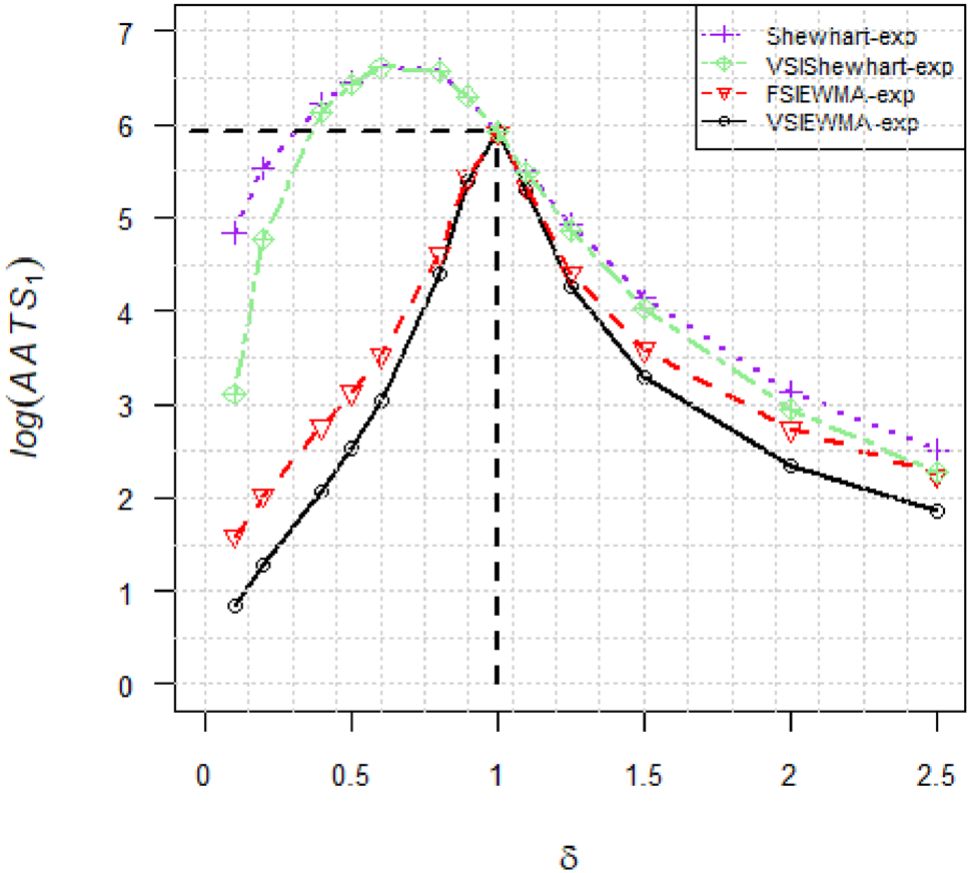
Curves of $A{ATS}_{1}$. The vertical axis is logarithmic for the sake of comparison.

To establish the superiority of the VSIEWMA-exp chart in shift detection, we conduct a numerical comparison with the three existing charts (Shewhart-exp, VSIShewhart-exp, and FSIEWMA-exp). The former two are Shewhart-type control charts using FSI and VSI schemes, respectively, while the latter is an EWMA-type control chart using the FSI scheme. Set $m=\left\{\mathrm{50,200,500},+\infty \right\}$, $N=100$ and $\delta =\{\mathrm{0.9,0.8,0.6,0.5,0.4,0.2,0.1,1.1,1.25,1.5,2},\mathrm{2.5,5},10\}$, where $\{\mathrm{0.9,0.8,0.6,0.5,0.4,0.2,0.1}\}$ and $\{\mathrm{1.1,1.25,1.5,2},\mathrm{2.5,5},10\}$ represent the downward and upward shifts, respectively. From Tables 1, 2, 3 and 4, we can conclude the following results:

1. As $m$ increases, the optimal model parameters and the ${AATS}_{1}$ are both gradually converge between the two cases of unknown ${\eta }_{0}$ ($m<+\infty$) and known ${\eta }_{0}$ ($m=+\infty$). Additionally, when the shift level is large, the difference in ${AATS}_{1}$ becomes small. This indicates that when $m$ and shift levels are significant, the impact of parameter estimation on control chart performance is relatively small.
2. Across various combinations of $(m,\delta )$, the proposed VSIEWMA-exp chart consistently exhibits the smallest ${AATS}_{1}$ values among competitive charts, suggesting superior sensitivity in detecting process shift.
3. The ${AATS}_{1}$ values of the proposed VSIEWMA-exp chart are consistently lower than the ${AATS}_{0}$ value, demonstrating unbiasedness. However, both Shewhart-exp and VSIShewhart-exp charts exhibit bias, as evident from the ${AATS}_{1}$ curve in Fig. 3.

**Table 1:** The values of ${AATS}_{1}$ with optimal model parameters for $m=50$ (in-control $A{ATS}_{0}=370.4$).

Optimal model parameters					$m=50$			
$\delta$	${\lambda }^{*}$	${K}^{*}$	${W}^{*}$	(${{h}_{1}}^{*},{{h}_{2}}^{*}$)	VSIEWMA-exp	FSIEWMA-exp	VSIShewhart-exp	Shewhart-exp
0.9	0.03	2.5628	0.5071	(2.5, 0.1)	325.02	332.56	500.59	501.24
0.8	0.03	2.5628	0.5071	(2.5, 0.1)	196.11	215.89	625.87	627.43
0.6	0.03	2.5622	0.5573	(2.3, 0.1)	32.90	51.61	687.91	691.95
0.5	0.06	2.7403	0.6218	(2.1, 0.1)	15.97	29.69	595.70	608.81
0.4	0.11	2.8526	0.6227	(2.1, 0.1)	9.18	18.99	446.41	492.93
0.2	0.26	2.9212	0.8070	(1.7, 0.1)	3.77	8.37	120.53	246.98
0.1	0.40	2.9064	0.9542	(1.5, 0.1)	2.39	5.20	23.10	123.74
1.1	1.00	2.7387	0.6218	(2.1,0.1)	258.51	262.85	258.51	262.85
1.25	0.95	2.7428	0.6218	(2.1, 0.1)	148.36	155.15	148.65	155.50
1.5	0.05	2.7014	0.5088	(2.5, 0.1)	44.04	57.18	65.30	71.73
2	0.10	2.8368	0.7017	(1.9, 0.1)	12.33	18.50	20.87	24.70
2.5	0.18	2.9071	0.9542	(1.5, 0.1)	7.07	10.49	10.44	12.87
5	0.55	2.8629	1.3668	(1.2, 0.1)	2.64	3.33	2.91	3.54
10	0.79	2.7801	1.6589	(1.1, 0.1)	1.64	1.85	1.67	1.87

**Table 2:** The values of ${AATS}_{1}$ with optimal model parameters for $m=200$ (in-control $A{ATS}_{0}=370.4$).

Optimal model parameters					$m=200$			
$\delta$	${\lambda }^{*}$	${K}^{*}$	${W}^{*}$	(${{h}_{1}}^{*},{{h}_{2}}^{*}$)	VSIEWMA-exp	FSIEWMA-exp	VSIShewhart-exp	Shewhart-exp
0.9	0.03	2.3798	0.6108	(2.1, 0.1)	277.02	287.15	530.16	530.47
0.8	0.03	2.3798	0.6108	(2.1, 0.1)	112.99	135.20	686.16	689.93
0.6	0.05	2.5519	0.5537	(2.3, 0.1)	22.52	37.76	721.56	723.88
0.5	0.08	2.6828	0.5846	(2.2, 0.1)	13.08	24.65	611.24	619.94
0.4	0.12	2.7690	0.6547	(2.0, 0.1)	8.22	16.75	451.37	497.85
0.2	0.28	2.8681	0.8673	(1.6, 0.1)	3.61	7.85	118.12	249.22
0.1	0.42	2.8672	1.0467	(1.4, 0.1)	2.35	4.98	22.21	124.86
1.1	0.94	2.7481	0.6545	(2.0, 0.1)	243.62	249.11	244.08	249.61
1.25	0.03	2.3798	0.6108	(2.1, 0.1)	91.36	105.63	133.53	141.41
1.5	0.05	2.5519	0.5537	(2.3, 0.1)	29.78	40.54	57.98	64.82
2	0.13	2.7852	0.7446	(1.8, 0.1)	10.90	16.18	19.26	23.12
2.5	0.19	2.8398	0.8670	(1.6, 0.1)	6.66	9.70	9.94	12.33
5	0.55	2.8413	1.3561	(1.2, 0.1)	2.59	3.27	2.85	3.50
10	0.81	2.7690	1.6458	(1.1, 0.1)	1.63	1.84	1.67	1.87

**Table 3:** The values of ${AATS}_{1}$ with optimal model parameters for $m=500$ (in-control $A{ATS}_{0}=370.4$).

Optimal model parameters					$m=500$			
$\delta$	${\lambda }^{*}$	${K}^{*}$	${W}^{*}$	(${{h}_{1}}^{*},{{h}_{2}}^{*}$)	VSIEWMA-exp	FSIEWMA-exp	VSIShewhart-exp	Shewhart-exp
0.9	0.03	2.3133	0.5472	(2.3, 0.1)	249.56	260.16	538.91	539.24
0.8	0.03	2.3133	0.5472	(2.3, 0.1)	93.12	114.42	704.57	705.43
0.6	0.06	2.5604	0.5822	(2.2, 0.1)	21.37	35.47	731.06	733.43
0.5	0.09	2.6751	0.5838	(2.2, 0.1)	12.65	23.58	617.23	625.20
0.4	0.13	2.7567	0.6538	(2.0, 0.1)	8.06	16.22	455.11	501.61
0.2	0.28	2.8545	0.8659	(1.6, 0.1)	3.61	7.72	118.37	251.08
0.1	0.42	2.8588	1.0451	(1.4, 0.1)	2.33	4.92	22.17	125.79
1.1	0.03	2.3178	0.6439	(2.0, 0.1)	227.73	235.42	240.80	246.54
1.25	0.03	2.3178	0.6439	(2.0, 0.1)	78.08	91.33	130.63	138.63
1.5	0.06	2.5604	0.5822	(2.2, 0.1)	27.86	37.87	56.77	63.61
2	0.14	2.7724	0.7435	(1.8, 0.1)	10.65	15.71	19.01	22.85
2.5	0.19	2.8204	0.8656	(1.6, 0.1)	6.55	9.53	9.85	12.25
5	0.55	2.8361	1.3539	(1.2, 0.1)	2.58	3.26	2.85	3.50
10	0.81	2.7685	1.6432	(1.1, 0.1)	1.63	1.84	1.67	1.87

**Table 4:** The values of ${AATS}_{1}$ with optimal model parameters for $m=+\infty$ (in-control $A{ATS}_{0}=370.4$).

Optimal model parameters					$m=+\infty$			
$\delta$	${\lambda }^{*}$	${K}^{*}$	${W}^{*}$	(${{h}_{1}}^{*},{{h}_{2}}^{*}$)	VSIEWMA-exp	FSIEWMA-exp	VSIShewhart-exp	Shewhart-exp
0.9	0.03	2.2510	0.5730	(2.2, 0.1)	220.45	229.92	545.63	545.98
0.8	0.03	2.2510	0.5730	(2.2, 0.1)	82.16	101.19	712.67	719.95
0.6	0.06	2.5209	0.6497	(2.0, 0.1)	20.58	33.73	738.54	740.96
0.5	0.09	2.6426	0.6519	(2.0, 0.1)	12.41	22.75	622.47	629.83
0.4	0.14	2.7494	0.6531	(2.0, 0.1)	7.95	15.80	458.53	505.08
0.2	0.28	2.8439	0.8650	(1.6, 0.1)	3.58	7.61	118.77	252.81
0.1	0.42	2.8525	1.0440	(1.4, 0.1)	2.32	4.88	22.18	126.66
1.1	0.03	2.2604	0.7824	(1.7, 0.1)	200.69	207.70	238.58	244.45
1.25	0.03	2.2532	0.6406	(2.0, 0.1)	70.86	82.26	128.77	136.85
1.5	0.06	2.5209	0.6497	(2.0, 0.1)	26.65	36.00	56.01	62.86
2	0.16	2.7756	0.7428	(1.8, 0.1)	10.53	15.36	18.85	22.69
2.5	0.19	2.8049	0.8647	(1.6, 0.1)	6.47	9.41	9.80	12.19
5	0.54	2.8345	1.3525	(1.2, 0.1)	2.57	3.26	2.85	3.50
10	0.81	2.7683	1.6415	(1.1, 0.1)	1.63	1.84	1.67	1.87

As we know, the mean of the transformed exponential data (${X}^{1/3.6}$) is ${u}_{0}={{\widehat{\eta }}_{0}}^{1/3.6}\Gamma \left(1+1/3.6\right)$, and the standard deviation is ${\sigma }_{0}={{\widehat{\eta }}_{0}}^{1/3.6}\sqrt{\left(\Gamma \left(1+2/3.6\right)-{\Gamma }^{2}\left(1+1/3.6\right)\right)}$. Based on the normality assumption with mean and standard deviation ${u}_{0}$ and ${\sigma }_{0}$, we developed an EWMA-type control chart with a VSI scheme (VSIEWMA-nor). In the case of $m=+\infty$, we have obtained the optimal model parameters for the VSIEWMA-nor chart at different shift levels $\delta$, and calculated the ${AATS}_{0}$ of the transformed data accordingly, denoted as ${AATS}_{0}\_N$, and display it in Table 5. We also provide the ${AATS}_{0}$ of the VSIEWMA-exp control chart, calculated based on the optimal model parameters from Table 4, denoted as ${AATS}_{0}\_E$, and display it in Table 5. It is observed that the $A{ATS}_{0}\_N$ is not equal to 370.4. Because the transformed data only approximates a normal distribution, not a normal distribution, it results in $A{ATS}_{0}\_N$ greater than 370. However, the ${AATS}_{0}$ of our proposed VSIEWMA-exp chart is 370.4, as the transformed data follows a Weibull distribution. Therefore, utilizing the proposed control chart is more reliable.

**Table 5:** Performance of the transformed data based on VSIEWMA-nor chart.

$\delta$	${\lambda }^{*}$	${K}^{*}$	${W}^{*}$	(${{h}_{1}}^{*},{{h}_{2}}^{*}$)	$A{ATS}_{0}\_N$	$A{ATS}_{0}\_E$
0.9	0.03	2.2699	0.5822	(2.1, 0.1)	370.25	370.4
0.8	0.03	2.2699	0.5822	(2.1, 0.1)	370.25	370.4
0.6	0.05	2.4705	0.5575	(2.2, 0.1)	372.65	370.4
0.5	0.09	2.6637	0.6280	(2.0, 0.1)	380.61	370.4
0.4	0.13	2.7649	0.6295	(2.0, 0.1)	391.58	370.4
0.2	0.27	2.9106	0.8378	(1.6, 0.1)	449.20	370.4
0.1	0.37	2.9497	1.0154	(1.4, 0.1)	509.98	370.4
1.1	0.03	2.2699	0.5822	(2.1, 0.1)	370.25	370.4
1.25	0.03	2.2699	0.5822	(2.1, 0.1)	370.25	370.4
1.5	0.06	2.5368	0.6255	(2.0, 0.1)	374.37	370.4
2	0.14	2.7826	0.7169	(1.8, 0.1)	394.79	370.4
2.5	0.22	2.8774	0.7726	(1.7, 0.1)	425.29	370.4
5	0.44	2.9684	1.3277	(1.2, 0.1)	567.42	370.4
10	0.68	2.9942	1.6330	(1.1, 0.1)	867.50	370.4

## Implementation of the proposed schemes

**Figure 4:**
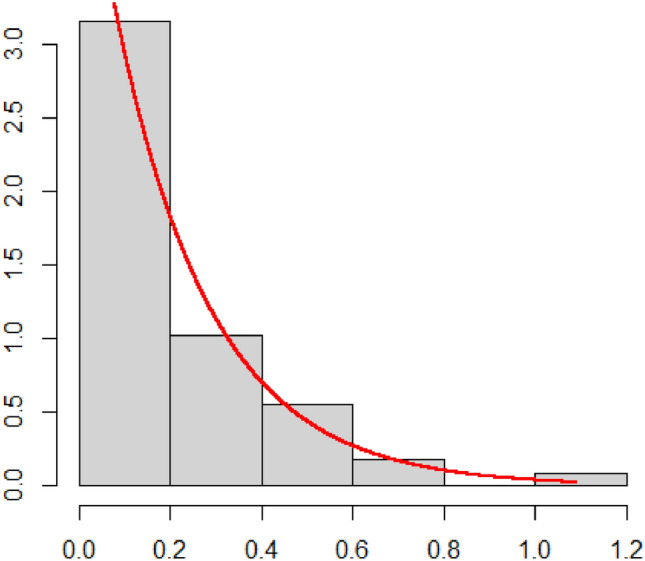
Histogram of Urinary Tract Infections rate.

In this section, a dataset of urinary tract infections (UTIs) is considered to demonstrate our proposed control chart, which is presented in Table 6 by Santiago et al.^31^. The purpose of this data is to monitor the changes in the infection rate of UTI, so the days in between discharge of males in nosocomial UTIs in patients is recorded. For more details, please refer to Santiago et al.^31^. It can be observed from Fig. 4 that this dataset exhibits significant characteristics of an exponential distribution. Moreover, the p-value of the K-S (Kolmogorov–Smirnov) test is 0.8112, indicating that this dataset follows the exponential distribution.

**Table 6:** The urinary tract infections (UTIs) dataset.

Phase I						Phase II			
$i$	Samples	$i$	Samples	$i$	Samples	$i$	Samples	$i$	Samples
1	0.57014	19	0.12014	37	0.27083	1	0.38231	19	0.30506
2	0.07431	20	0.11458	38	0.04514	2	0.44270	20	0.25665
3	0.15278	21	0.00347	39	0.13542	3	0.28831		
4	0.14583	22	0.12014	40	0.08681	4	0.18758		
5	0.13889	23	0.04861	41	0.40347	5	0.09674		
6	0.14931	24	0.02778	42	0.12639	6	0.20090		
7	0.03333	25	0.32639	43	0.18403	7	0.57208		
8	0.08681	26	0.64931	44	0.70833	8	0.49713		
9	0.33681	27	0.14931	45	0.15625	9	0.13790		
10	0.03819	28	0.01389	46	0.24653	10	0.31507		
11	0.24653	29	0.03819	47	0.04514	11	0.74995		
12	0.29514	30	0.46806	48	0.01736	12	0.09923		
13	0.11944	31	0.22222	49	1.08889	13	0.49534		
14	0.05208	32	0.29514	50	0.05208	14	0.68715		
15	0.12500	33	0.53472	51	0.02778	15	0.24903		
16	0.25000	34	0.15139	52	0.03472	16	0.22786		
17	0.40069	35	0.52569	53	0.23611	17	0.13581		
18	0.02500	36	0.07986	54	0.35972	18	0.63413

**Figure 5:**
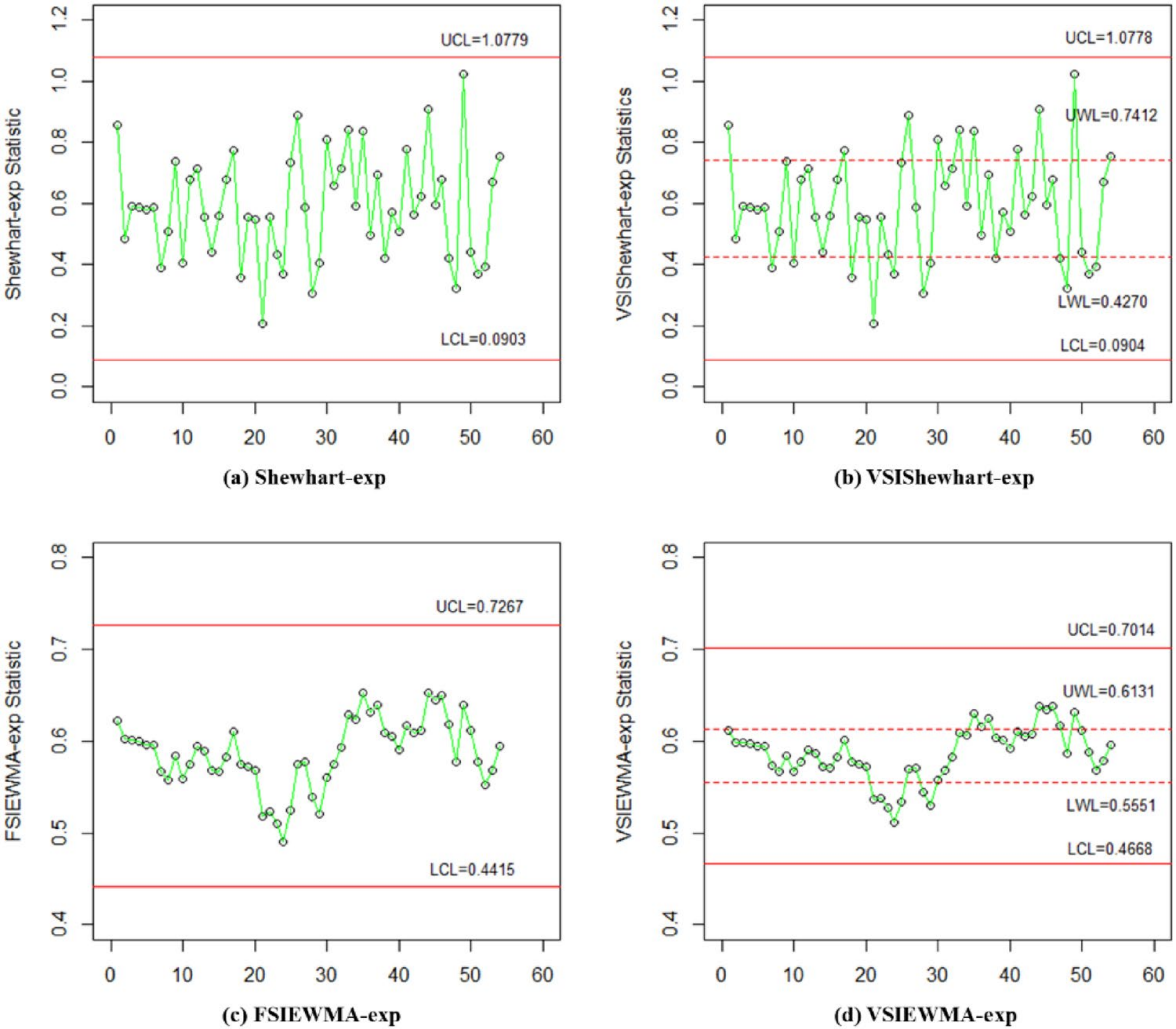
The VSIEWMA-exp control chart for phase I dataset.

Firstly, we use in-control Phase I data to estimate ${\eta }_{0}$. Subsequently, we apply the optimization algorithm to obtain optimal model parameters for calculating the control and warning limits. Figure 5 displays the plotted points for the Phase I data. It can be observed that none of the control charts show any false alarms. It is consistent with Santiago et al.^31^, indicating that they can be utilized to monitor Phase II data.

**Figure 6:**
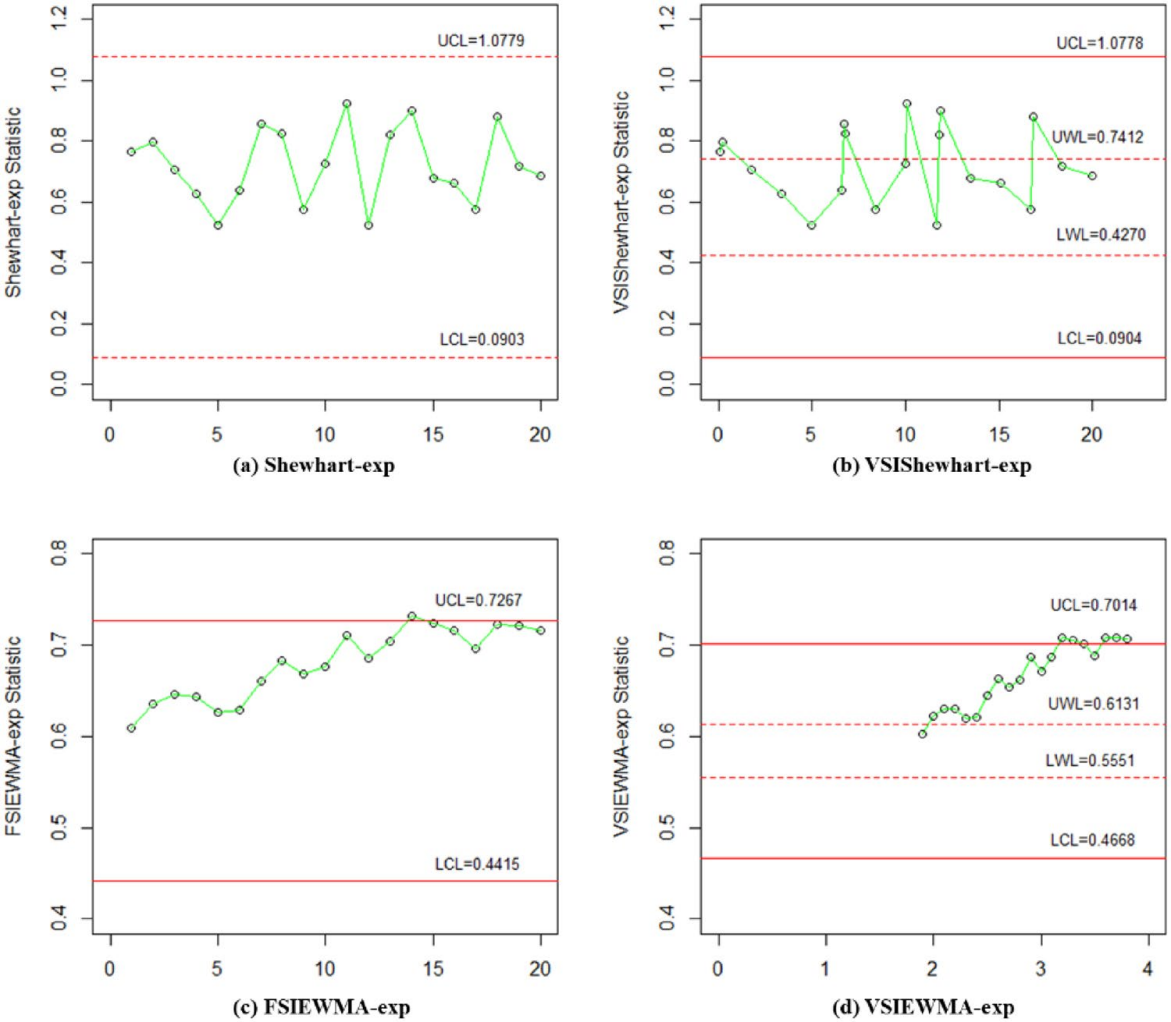
The VSIEWMA-exp control chart for phase II dataset.

Figure 6 shows the detection results that the process is out-of-control. Regarding detection capability, Shewhart-exp and VSIShewhart-exp control charts failed to detect shifts. The FSIEWMA-exp chart identifies only one out-of-control sample, while the proposed VSIEWMA-exp chart detects five samples. Regarding detection efficiency, only the first example falls within the central region, while the others all fall within the warning region. This suggests that a shorter sampling interval is employed after the first example. Consequently, the proposed VSIEWMA-exp chart takes less time to trigger an out-of-control signal than the FSIEWMA-exp chart.

## Summary remarks

The purpose of this study is to propose a VSIEWMA-exp control chart. The theoretical method is to use the VSI scheme to construct an EWMA-type control chart for the exponential distribution and consider the situation of unknown parameter. In addition, we use the Markov chain approach to propose two performance measures for the proposed control chart, $CATS$ and $AATS$. We also provide an optimization algorithm for model parameters. In Section "Comparison of proposed and existing schemes", we compared our proposed control chart with the other three control charts, and the results showed that our proposed control chart outperformed other competitors in most cases. Finally, the proposed control chart is demonstrated using the UTI data as an example, and the results showed that the proposed control chart has high efficiency in monitoring the Phase II data in this case.

It is worth noting that this study only used the VSI scheme. Future research can consider monitoring statistical data $\overline{X }$ based on subgroup sample size. At this point, $\overline{X }$ follows a gamma distribution, and EWMA control charts designed by VSS or VSI can be considered.

## Supplementary Information

Supplementary Information.

## Data Availability

All data generated or analyzed during this study are included in this published article.
