# Supplementary material for: An enhanced EWMA chart with variable sampling interval scheme for monitoring the exponential process with estimated parameter
Source: Sci Rep. 2024 Apr 4;14:7958. doi: 10.1038/s41598-024-58675-7 (PMC11371916; doi:10.1038/s41598-024-58675-7)
Supplement: Supplementary file 1 — Supplementary Information. [file 41598_2024_58675_MOESM1_ESM.docx]

### $m=+\infty$

###All the values of K and W based on the given values of $A{ATS}_{0}$

###K

la=seq(0.03,1,0.01);h1=seq(1.1,2.5,0.1);h2=seq(0.1,0.9,0.1) #############$h1=h_{1}$; $h2=h_{2}$; $la=\lambda$

N=100;i=1:(2*N+1)

library(doParallel)

cl<-makeCluster(6)

registerDoParallel(cl)

K<-

foreach(v=1:length(h2))%:%

foreach(q=1:length(h1))%:%

foreach(s=1:length(la))%dopar%{

fun<-function(k){

N=100;i=1:(2*N+1)

l1=0.9011018-k*sqrt(0.8892902-0.81198445)

u1=0.9011018+k*sqrt(0.8892902-0.81198445)

lcl=0.9011018-k*sqrt((la[s]/(2-la[s]))*(0.8892902-0.81198445))

ucl=0.9011018+k*sqrt((la[s]/(2-la[s]))*(0.8892902-0.81198445))

fr<-function(w){

l2=0.9011018-w*sqrt(0.8892902-0.81198445)

u2=0.9011018+w*sqrt(0.8892902-0.81198445)

p1=(pweibull(u2,scale=1,shape=3.6)-pweibull(l2,scale=1,shape=3.6))/(pweibull(u1,scale=1,shape=3.6)-pweibull(l1,scale=1,shape=3.6))

p2=1-p1

p1*h1[q]+p2*h2[v]-1

}

w=uniroot(fr,c(0,2))$root

lwl=0.9011018-w*sqrt((la[s]/(2-la[s]))*(0.8892902-0.81198445))

uwl=0.9011018+w*sqrt((la[s]/(2-la[s]))*(0.8892902-0.81198445))

d=(ucl-lcl)/(2*N+1)

n1=round((lwl-lcl)/d);n2=2*N+1-2*n1

pij=function(i,j){

mi=lcl+(i-1)*(ucl-lcl)/(2*N+1)+(lcl+i*(ucl-lcl)/(2*N+1)-(lcl+(i-1)*(ucl-lcl)/(2*N+1)))/2

pweibull((lcl+j*(ucl-lcl)/(2*N+1)-(1-la[s])*mi)/la[s],scale=1,shape=3.6)-pweibull((lcl+(j-1)*(ucl-lcl)/(2*N+1)-(1-la[s])*mi)/la[s],scale=1,shape=3.6)

}

P<-outer(i,i,pij)

m1=matrix(c(rep(h2[v],n1),rep(h1[q],n2),rep(h2[v],n1)),2*N+1,1);p0=matrix(c(rep(0,N),1,rep(0,N)),1,2*N+1)

I=diag(1,2*N+1,2*N+1);SP=solve(I-P)

ATS0=p0%*%SP%*%m1

ats0=ATS0[1,1]

ats0-370.4

}

uniroot(fun,c(2,4))$root

}

stopCluster(cl)

K=array(unlist(K),dim=c(length(la),length(h1),length(h2)))

###W

W=array(0,dim=c(length(la),length(h1),length(h2)))

for(v in 1:length(h2)){

for(q in 1:length(h1)){

for(s in 1:length(la)){

fr<-function(w){

l1=0.9011018-K[s,q,v]*sqrt(0.8892902-0.81198445)

u1=0.9011018+K[s,q,v]*sqrt(0.8892902-0.81198445)

l2=0.9011018-w*sqrt(0.8892902-0.81198445)

u2=0.9011018+w*sqrt(0.8892902-0.81198445)

p1=(pweibull(u2,scale=1,shape=3.6)-pweibull(l2,scale=1,shape=3.6))/(pweibull(u1,scale=1,shape=3.6)-pweibull(l1,scale=1,shape=3.6))

p2=1-p1

p1*h1[q]+p2*h2[v]-1

}

W[s,q,v]=uniroot(fr,c(0,2))$root

}

}

}

###Optimized model parameters $\left( \lambda^{*},K^{*},W^{*},{h_{1}}^{*},{h_{2}}^{*} \right)$ and the corresponding minimum ${AATS}_{1}$ based on $\delta=\{0.1,0.2,0.4,0.6,0.8,0.9,1.1,1.25,1.5,2,2.5,5,10\}$

library(doParallel)

cl<-makeCluster(8)

registerDoParallel(cl)

ats<-

foreach(v=1:length(h2))%:%

foreach(q=1:length(h1))%:%

foreach(s=1:length(la))%dopar%{

del=0.9 ###################$del=\delta$, it can choose the values of {0.1,0.2,0.4,0.6,0.8,0.9,1.1,1.25,1.5,2,2.5,5,10}

N=100;i=1:(2*N+1)

lcl=0.9011018-K[s,q,v]*sqrt((la[s]/(2-la[s]))*(0.8892902-0.81198445))

ucl=0.9011018+K[s,q,v]*sqrt((la[s]/(2-la[s]))*(0.8892902-0.81198445))

lwl=0.9011018-W[s,q,v]*sqrt((la[s]/(2-la[s]))*(0.8892902-0.81198445))

uwl=0.9011018+W[s,q,v]*sqrt((la[s]/(2-la[s]))*(0.8892902-0.81198445))

d=(ucl-lcl)/(2*N+1)

n1=round((lwl-lcl)/d);n2=2*N+1-2*n1

pij=function(i,j){

lj=lcl+((j-1)*(ucl-lcl))/(2*N+1);uj=lcl+(j*(ucl-lcl))/(2*N+1)

li=lcl+((i-1)*(ucl-lcl))/(2*N+1);ui=lcl+(i*(ucl-lcl))/(2*N+1)

mi=(li+ui)/2

pweibull(((1/del)^(1/3.6))*(uj-(1-la[s])*mi)/la[s],scale=1,shape=3.6)-pweibull(((1/del)^(1/3.6))*(lj-(1-la[s])*mi)/la[s],scale=1,shape=3.6)

}

P<-outer(i,i,pij)

m1=matrix(c(rep(h2[v],n1),rep(h1[q],n2),rep(h2[v],n1)),2*N+1,1);p0=matrix(c(rep(0,N),1,rep(0,N)),1,2*N+1)

I=diag(1,2*N+1,2*N+1);SP=solve(I-P)

ATS1=p0%*%SP%*%m1

ATS1[1,1]

}

stopCluster(cl)

ATS1=array(unlist(ats),dim=c(length(la),length(h1),length(h2)))

min_value=min(ATS1)

min_index=which(ATS1 == min_value, arr.ind = TRUE)

min_value ###################The minimum $A{ATS}_{1}$

min_index ########## The indices correspond to the optimal parameter K and W. The indices corresponding to the optimal $\lambda$, $h_{1}$, and $h_{2}$ are the first, second, and third, respectively.

### $m<+\infty$, i.e., ($m=50,200,500$)

###All the values of K and W based on the given values of $A{ATS}_{0}$

###K

rm(list=ls(all=TRUE))

la=seq(0.03,1,0.01);h1=seq(1.1,2.5,0.1);h2=0.1 ###Note that h2 is set to 0.1, because when $m=+\infty$, no matter what value $\delta$ takes, the optimal h2 is always 0.1. For saving the computational time, $h2=0.1$.

m=50### It can choose the values of {$50,200,500$}.

N=100;i=1:(2*N+1)

library(doParallel)

cl<-makeCluster(8)

registerDoParallel(cl)

K<-

foreach(q=h1,.combine=cbind)%:%

foreach(s=la,.combine=c)%dopar%{

fun<-function(k){

l1=0.9011018-k*sqrt(0.8892902-0.81198445)

u1=0.9011018+k*sqrt(0.8892902-0.81198445)

lcl=0.9011018-k*sqrt((s/(2-s))*(0.8892902-0.81198445))

ucl=0.9011018+k*sqrt((s/(2-s))*(0.8892902-0.81198445))

fr<-function(w){

fr1<-function(z){

l1=0.9011018-k*sqrt(0.8892902-0.81198445)

u1=0.9011018+k*sqrt(0.8892902-0.81198445)

l2=0.9011018-w*sqrt(0.8892902-0.81198445)

u2=0.9011018+w*sqrt(0.8892902-0.81198445)

p1=(pweibull(z*u2,scale=1,shape=3.6)-pweibull(z*l2,scale=1,shape=3.6))/(z*pweibull(u1,scale=1,shape=3.6)-pweibull(z*l1,scale=1,shape=3.6))

p2=1-p1

(p1*q+p2*h2)*dgamma(z,shape=m,scale=1/m)

}

integrate(Vectorize(fr1),lower=0,upper=2)$value-1

}

w=uniroot(fr,c(0,2))$root

lwl=0.9011018-w*sqrt((s/(2-s))*(0.8892902-0.81198445))

uwl=0.9011018+w*sqrt((s/(2-s))*(0.8892902-0.81198445))

d=(ucl-lcl)/(2*N+1)

n1=round((lwl-lcl)/d);n2=2*N+1-2*n1

fr2<-function(z){

pij=function(i,j){

lj=lcl+((j-1)*(ucl-lcl))/(2*N+1);uj=lcl+(j*(ucl-lcl))/(2*N+1)

li=lcl+((i-1)*(ucl-lcl))/(2*N+1);ui=lcl+(i*(ucl-lcl))/(2*N+1)

mi=(li+ui)/2

pweibull(((z/del)^(1/3.6))*(uj-(1-s)*mi)/s,scale=1,shape=3.6)-pweibull(((z/del)^(1/3.6))*(lj-(1-s)*mi)/s,scale=1,shape=3.6)

}

P<-outer(i,i,pij)

m1=matrix(c(rep(h2,n1),rep(q,n2),rep(h2,n1)),2*N+1,1);p0=matrix(c(rep(0,N),1,rep(0,N)),1,2*N+1)

I=diag(1,2*N+1,2*N+1);SP=solve(I-P)

ATS0=p0%*%SP%*%m1

ats0=ATS0[1,1]

ats0*dgamma(z,shape=m,scale=1/m)

}

integrate(Vectorize(fr2),lower=0,upper=2)$value-370.4

}

uniroot(fun,c(2,4))$root

}

stopCluster(cl)

###W

W=array(0,dim=c(length(la),length(h1)))

for(q in 1:length(h1)){

for(s in 1:length(la)){

fr<-function(w){

fr1<-function(z){

l1=0.9011018-K[s,q]*sqrt(0.8892902-0.81198445)

u1=0.9011018+K[s,q]*sqrt(0.8892902-0.81198445)

l2=0.9011018-w*sqrt(0.8892902-0.81198445)

u2=0.9011018+w*sqrt(0.8892902-0.81198445)

p1=(pweibull(u2,scale=1,shape=3.6)-pweibull(l2,1,shape=3.6))/(pweibull(u1,scale=1,shape=3.6)-pweibull(l1,scale=1,shape=3.6))

p2=1-p1

(p1*h1[q]+p2*h2)*dgamma(z,shape=m,scale=1/m)

}

integrate(Vectorize(fr1),lower=0,upper=2)$value-1

}

W[s,q]=uniroot(fr,c(0,2))$root

}

}

###Optimized model parameters $\left( \lambda^{*},K^{*},W^{*},{h_{1}}^{*},{h_{2}}^{*} \right)$ and the corresponding minimum ${AATS}_{1}$ based on $\delta=\{0.1,0.2,0.4,0.6,0.8,0.9,1.1,1.25,1.5,2,2.5,5,10\}$

del=0.9###################$del=\delta$, it can choose the values of {0.1,0.2,0.4,0.6,0.8,0.9,1.1,1.25,1.5,2,2.5,5,10}

library(doParallel)

cl<-makeCluster(8)

registerDoParallel(cl)

aats1<-

foreach(q=1:ncol(K),.combine=cbind)%:%

foreach(s=1:nrow(K),.combine=c)%dopar%{

lcl=0.9011018-K[s,q]*sqrt((la[s]/(2-la[s]))*(0.8892902-0.81198445))

ucl=0.9011018+K[s,q]*sqrt((la[s]/(2-la[s]))*(0.8892902-0.81198445))

lwl=0.9011018-W[s,q]*sqrt((la[s]/(2-la[s]))*(0.8892902-0.81198445))

uwl=0.9011018+W[s,q]*sqrt((la[s]/(2-la[s]))*(0.8892902-0.81198445))

d=(ucl-lcl)/(2*N+1)

n1=round((lwl-lcl)/d);n2=2*N+1-2*n1

fr2<-function(z){

pij=function(i,j){

lj=lcl+((j-1)*(ucl-lcl))/(2*N+1);uj=lcl+(j*(ucl-lcl))/(2*N+1)

li=lcl+((i-1)*(ucl-lcl))/(2*N+1);ui=lcl+(i*(ucl-lcl))/(2*N+1)

mi=(li+ui)/2

pweibull(((z/del)^(1/3.6))*(uj-(1-la[s])*mi)/la[s],scale=1,shape=3.6)-pweibull(((z/del)^(1/3.6))*(lj-(1-la[s])*mi)/la[s],scale=1,shape=3.6)

}

P<-outer(i,i,pij)

m1=matrix(c(rep(h2,n1),rep(h1[q],n2),rep(h2,n1)),2*N+1,1);p0=matrix(c(rep(0,N),1,rep(0,N)),1,2*N+1)

I=diag(1,2*N+1,2*N+1);SP=solve(I-P)

ATS1=p0%*%SP%*%m1

ats1=ATS1[1,1]

ats1*dgamma(z,shape=m,scale=1/m)

}

integrate(Vectorize(fr2),lower=0,upper=2)$value

}

stopCluster(cl)

min_value=min(aats1)

min_index=which(aats1 == min_value, arr.ind = TRUE)

min_value ###################The minimum $A{ATS}_{1}$

min_index ##########The indices correspond to the optimal parameter K and W. The indices corresponding to the optimal $\lambda$ and $h_{1}$ are the first and second, respectively.
